# Health-related quality of life and psychosocial outcomes after treatment for congenital heart disease among school-aged children and adolescents in China: an online cross-sectional comparative study

**DOI:** 10.3389/fped.2026.1878766

**Published:** 2026-08-05

**Authors:** Yuyang Huang, Weizhi Zhang, Dongping Li, Haojie Zhou, Hanyi Yao, Zhangqing Yi, Zehong Wu, Jiaming Wu

**Affiliations:** Second Xiangya Hospital, Central South University, Changsha, China

**Keywords:** cardiac rehabilitation, congenital heart disease, depressive symptoms, health-related quality of life, PedsQL, self-esteem

## Abstract

**Background:**

As survival after treatment for congenital heart disease (CHD) continues to improve, long-term outcomes beyond survival have become increasingly important. Evidence from mainland China remains limited, particularly for school-aged children and adolescents after treatment for CHD. This study evaluated health-related quality of life (HRQoL), depressive symptoms, and self-esteem in this population.

**Methods:**

This online cross-sectional comparative study included children and adolescents aged 7–18 years who had undergone surgical or minimally invasive treatment for CHD at least 6 months previously and controls of comparable age without known CHD or other reported chronic medical conditions. HRQoL was assessed using the Pediatric Quality of Life Inventory 4.0 Generic Core Scales (PedsQL 4.0), depressive symptoms using the Center for Epidemiologic Studies Depression Scale for Children (CES-DC), and self-esteem using the Rosenberg Self-Esteem Scale (RSES). Continuous variables were summarized as median (interquartile range, IQR), and between-group comparisons were performed using non-parametric tests. Age- and sex-adjusted linear regression models were fitted for the major outcomes.

**Results:**

A total of 78 post-treatment CHD patients and 90 controls were included. Compared with controls, post-treatment CHD patients had lower PedsQL 4.0 scores across Physical, Emotional, Social, and School Functioning domains, as well as Physical Health, Psychosocial Health, and Total Scores (all *P* < 0.001). In the age- and sex-adjusted model, controls had a higher PedsQL Total Score than post-treatment CHD patients (B = 13.31, 95% CI: 8.25–18.36, *P* < 0.001). No statistically significant difference in CES-DC scores was observed between groups [10.5 (3.25–17.75) vs. 8.0 (4.0–14.0), *P* = 0.285]. RSES scores were lower in post-treatment CHD patients than in controls [31.0 (28.0–36.75) vs. 36.0 (30.0–39.0), *P* = 0.001]. The adjusted model also showed higher RSES scores among controls (B = 2.83, 95% CI: 1.17–4.48, *P* = 0.001).

**Conclusion:**

In this online sample, school-aged children and adolescents after treatment for CHD reported poorer HRQoL and lower global self-esteem scores than controls without known CHD. No statistically significant difference in depressive symptom scores was observed. These findings support post-treatment care models that extend beyond cardiac status and incorporate functional recovery, school participation, family education, physical activity guidance, and psychosocial screening.

## Introduction

Congenital heart disease (CHD) is among the most common birth defects, affecting nearly 1% of live births worldwide (1). In China, more than 40,000 children and adolescents underwent treatment for CHD in 2021 alone (2). Advances in diagnosis, surgery, minimally invasive treatment, and perioperative care mean that increasing numbers of affected children now survive into adolescence and adulthood (3). For many patients, uneventful recovery after treatment permits return to school and social activities within several months (4). However, survival and anatomical repair do not necessarily imply full recovery in everyday life. Some children experience school absence, poorer academic performance, activity restriction, changes in appearance, or protective attitudes from parents and teachers (5, 6). These experiences may influence HRQoL and psychosocial adjustment long after the immediate treatment period.

Focusing specifically on children and adolescents after treatment for CHD is clinically important because this group represents a growing survivor population whose needs may differ from those of children with unrepaired CHD. Post-treatment outcomes can vary by lesion type, treatment strategy, residual disease, age at treatment, and time since treatment (7, 8). Recent studies continue to indicate that children and adolescents with CHD may experience impairments in HRQoL and psychosocial functioning, although evidence concerning school-aged post-treatment populations in mainland China remains limited (6, 7, 9, 10).

Cultural and educational contexts may also shape how children and families experience illness and recovery (6). Academic demands, family involvement in schooling, and perceptions of physical vulnerability may influence HRQoL, school reintegration, and self-evaluation in Chinese children after treatment for CHD. Findings from Western populations may therefore not be directly generalizable to mainland China. Against this background, we conducted an online cross-sectional comparative study to examine HRQoL, depressive symptoms, and self-esteem among school-aged children and adolescents after treatment for CHD in mainland China, using controls without known CHD as the comparison group.

## Materials & methods

### Study design and participants

This online cross-sectional comparative study was conducted using Sojump, a web-based survey platform, to distribute and collect questionnaire data. Eligible post-treatment participants were school-aged children and adolescents aged 7–18 years who had undergone surgical or minimally invasive treatment for CHD at least 6 months previously. Controls of comparable age without known CHD or other reported chronic medical conditions were recruited as the comparison group. No formal individual matching procedure was performed.

Post-treatment CHD group. Eligible participants were children and adolescents who were at least 6 months after surgical or minimally invasive treatment for CHD and had not been readmitted or undergone reoperation for major post-treatment complications. Candidates were recruited through WeChat-based post-treatment follow-up groups linked to the authors' institution, and clinical information, including diagnosis, treatment strategy, treatment date, and age at treatment, was obtained from the Hospital Information System (HIS) when available. To minimize potential confounding from structured rehabilitation interventions, participants with a history of cardiac rehabilitation were excluded. Additional exclusion criteria included (1) age outside 7–18 years or less than 6 months after treatment; (2) major post-treatment events within 6 months after treatment, uncontrolled cardiovascular or respiratory comorbidities, or clinically significant residual lesions requiring long-term treatment, hospitalization, or reintervention; and (3) other congenital anomalies, diagnosed psychiatric or cognitive disorders, inability to understand or complete the questionnaire, or lack of guardian consent.

Control group. Controls aged 7–18 years were recruited through social media platforms. Exclusion criteria included a self-reported history of congenital or acquired cardiovascular disease or cardiac surgery, recent or chronic illnesses requiring long-term management or hospitalization, psychiatric or cognitive disorders, inability to understand or complete the questionnaire, or lack of guardian consent. Because controls were identified from reported health history rather than clinical examination or echocardiographic screening, they are described as controls without known CHD rather than clinically verified healthy participants.

To support eligibility verification and data quality, the questionnaire included a brief personal-information section that allowed cross-checking with HIS for the post-treatment CHD group. HIS was also used to identify and exclude patients with major post-treatment complications or reinterventions. For controls, demographic characteristics and reported health history were collected to screen for chronic or cardiovascular conditions.

### Recruitment and data collection

Recruitment was conducted using online advertisements. For the post-treatment CHD group, an invitation poster was distributed in WeChat-based post-treatment follow-up groups and included the study purpose, eligibility criteria, and a QR code link to the Sojump questionnaire. For the control group, the invitation poster and QR code were disseminated primarily through social media platforms. Parents or legal guardians were first contacted through the recruitment channels and were provided with an online information sheet describing the study purpose, eligibility criteria, voluntary nature of participation, and data-confidentiality measures. After electronic informed consent had been obtained from the parent or legal guardian, children and adolescents completed the questionnaires either independently or, when necessary, with assistance from their guardian to understand the survey procedure. Children and adolescents were also asked to provide assent before completing the questionnaire. Online quality-control procedures included mandatory responses for core questionnaire items, eligibility screening before analysis, review of completion time, and exclusion of responses with inconsistent age, treatment history, or health-history information.

In this study, “interventional procedure” referred to therapeutic minimally invasive occlusion procedures for simple CHD, including percutaneous transcatheter closure and perventricular device closure via a lower partial sternotomy/right ventricular puncture. These procedures were distinct from diagnostic catheterization, right-heart catheterization, or coronary angiography. “Cardiac surgery with CPB” referred to open cardiac surgery performed with cardiopulmonary bypass.

The sample-size calculation was used as a planning reference rather than as evidence of population representativeness, given the online non-probability sampling design. Assuming a medium effect size (Cohen's d = 0.5), a two-sided significance level of 0.05, and 80% power, G*Power indicated that at least 67 participants per group would be required. To allow for incomplete or invalid questionnaires and potential comprehension issues among younger school-aged children, we aimed to recruit at least 75 respondents per group.

### Measures

(1) Pediatric Quality of Life 4.0 Generic Core Scale (PedsQL 4.0) (11)

The Pediatric Quality of Life 4.0 generic core scales include 23 items across 4 dimensions: Physical Functioning (8 items), Emotional Functioning (5 items), Social Functioning (5 items), and School Functioning (5 items). Participants rated the degree or frequency of each item on a 5-point Likert scale from 0 to 4, where 0 = never a problem, 1 = almost never a problem, 2 = sometimes a problem, 3 = often a problem, 4 = almost always a problem.

We used the Chinese version of the PedsQL 4.0 generic core scale. Item scores were reverse-scored and linearly transformed to a 0–100 scale (0 = 100, 1 = 75, 2 = 50, 3 = 25, 4 = 0), with higher scores indicating better HRQoL. Scale scores were calculated as the mean of the items in each domain, and the Total Score was calculated as the mean of all 23 items. The Psychosocial Health Summary Score was derived from the Emotional, Social, and School Functioning domains.

(2) Center for Epidemiologic Studies Depression Scale for Children (CES-DC) (12).

Depressive symptoms were assessed using the CES-DC, a widely used 20-item self-report screening instrument for children and adolescents. The CES-DC asks respondents to rate symptoms experienced during the past week on a 4-point scale from 0 (“not at all”) to 3 (“a lot”), and positively worded items were reverse-scored according to the standard scoring procedure. Total scores range from 0 to 60, with higher scores indicating greater depressive symptom burden. A total score of 16 or higher is commonly used as a screening threshold for elevated depressive symptoms rather than as a diagnosis of clinical depression.

(3) Rosenberg Self-Esteem Scale (RSES) (13, 14).

Self-esteem was assessed using a Chinese-language version of the 10-item Rosenberg Self-Esteem Scale (RSES), originally developed by Rosenberg and previously evaluated in Chinese children (13, 14). Each item was rated on a 4-point scale from 1 (“strongly agree”) to 4 (“strongly disagree”). In the questionnaire administered in this study, item 2 was worded negatively (“I do not feel that I have many good qualities”), rather than using the standard positive wording. Scoring therefore followed the wording actually presented: positively worded items 1, 4, 6, and 7 were reverse-scored, whereas negatively worded items 2, 3, 5, 8, 9, and 10 retained their original values before summation. Total scores ranged from 10 to 40, with higher scores indicating higher global self-esteem. Because no universally accepted cutoff has been established for Chinese children and adolescents using this scoring format, RSES scores were analyzed as continuous measures and interpreted comparatively rather than classified into low, moderate, or high self-esteem categories.

### CHD complexity classification

CHD anatomic complexity was categorized as simple, moderate-complexity, or complex CHD according to the 2020 European Society of Cardiology (ESC) guideline for the management of adult congenital heart disease (15). Although this framework was developed for adult congenital heart disease, it provides a pragmatic lesion-complexity structure and was used here to support exploratory stratification in a pediatric post-treatment sample. Simple CHD included isolated or minor shunt-type anomalies, such as atrial septal defect (ASD), ventricular septal defect (VSD), and patent ductus arteriosus (PDA). Complex CHD included major structural abnormalities or conditions associated with marked hemodynamic burden or pulmonary vascular disease. Moderate-complexity CHD was defined as disease complexity between these categories.

### Statistical analysis

Statistical analyses were performed using IBM SPSS Statistics (version 27.0; IBM Corp., Armonk, NY, USA). Normality of continuous variables was assessed using the Shapiro–Wilk test. Because most continuous variables were not normally distributed, they were summarized as median (IQR), whereas categorical variables were presented as frequencies and percentages. Between-group comparisons were performed using the Mann–Whitney *U*-test for continuous variables and the chi-square test for categorical variables, as appropriate. For comparisons across CHD complexity groups, the Kruskal–Wallis test was used.

The internal consistency of the PedsQL 4.0, CES-DC, and RSES in the current sample was evaluated using Cronbach's alpha coefficients. Multivariable linear regression analyses were performed for the three major outcomes, namely PedsQL Total Score, CES-DC total score, and RSES total score. Group status, age, and sex were included as covariates in the primary adjusted models. Regression coefficients are reported as unstandardized B coefficients with 95% confidence intervals. Spearman correlation analyses were performed within the post-treatment CHD group to explore associations between post-treatment interval and PedsQL Total Score, CES-DC total score, and RSES total score. Other potentially relevant confounders, including socioeconomic status, parental education, family functioning, geographic region, and physical activity level, were not available for adjustment. Post-treatment duration was not included in the between-group regression models because it was applicable only to the post-treatment CHD group; its associations with the major outcomes were explored separately using Spearman correlation analyses.

Given the limited sample size, especially in the complex CHD subgroup, subgroup analyses by treatment strategy, CHD anatomic complexity, and age at treatment were considered exploratory. The age-at-treatment cutoff of 7 years was selected to distinguish children who received treatment before school age from those who received treatment during school age, when school attendance and peer reintegration may be more directly affected. All tests were two-sided, and *P* < 0.05 was considered statistically significant.

## Results

### Participant flow and baseline characteristics

A total of 130 questionnaire responses were received from the post-treatment CHD group (Group 1, G1) and 119 from the control group (Group 2, G2). In G1, 52 responses were excluded for not meeting the eligibility criteria (age <7 or >18 years, or <6 months after treatment), leaving 78 participants for analysis. In G2, 29 responses were excluded because of ineligibility (not meeting the age requirement, unclear health/treatment history, or a reported history of cardiovascular disease or cardiac surgery), leaving 90 participants for analysis.

Table 1 summarizes the demographic characteristics of the study participants. The median age of the 78 post-treatment CHD participants was 9.0 years (IQR: 8.0–11.8), and 47 (60.3%) were male. The 90 controls had a median age of 10.0 years (IQR: 9.0–12.0), and 43 (47.8%) were male. No statistically significant differences in age or sex distribution were observed between groups (*P* = 0.152 and *P* = 0.106, respectively).

**Table 1:** Demographic and clinical characteristics of post-treatment CHD patients and controls without known CHD.

	Post-treatment CHD (*N* = 78)	Controls without known CHD (*N* = 90)	*P* value
	N (%) or median (IQR)	N (%) or median (IQR)	
Age (years)	9.00 (8.00–11.75)	10.00 (9.00–12.00)	0.152
Sex			
Male	47 (60.3%)	43 (47.8%)	0.106
Female	31 (39.7%)	47 (52.2%)	
Questionnaire completion time (seconds)	405.00 (282.25–532.25)	352.00 (248.25–463.75)	0.140
Age at treatment (years)	3.50 (2.00–8.00)	–	–
Post-treatment interval (months)	50.00 (26.00–73.25)	–	–
Treatment strategy		–	–
Minimally invasive occlusion procedure	33 (42.3%)	–	–
Cardiac surgery with cardiopulmonary bypass (CPB)	45 (57.7%)	–	–
CHD anatomic complexity			
Simple CHD	27 (34.6%)	–	–
Moderate-complexity CHD	41 (52.6%)	–	–
Complex CHD	10 (12.8%)	–	–
Primary diagnosis		–	–
VSD	38 (48.7%)	–	–
ASD	16 (20.5%)	–	–
PDA	0 (0.0%)	–	–
TOF	4 (5.1%)	–	–
DORV	5 (6.4%)	–	–
Other CHD	15 (19.3%)	–	–

Data are presented as *n* (%) for categorical variables and median (interquartile range) for continuous variables. Minimally invasive occlusion procedures included percutaneous transcatheter closure and perventricular device closure through a lower partial sternotomy with right ventricular puncture; these were therapeutic procedures rather than diagnostic catheterization. ASD, atrial septal defect; CHD, congenital heart disease; CPB, cardiopulmonary bypass; DORV, double-outlet right ventricle; IQR, interquartile range; PDA, patent ductus arteriosus; TOF, tetralogy of Fallot; VSD, ventricular septal defect.

In the post-treatment CHD group, the median age at treatment was 3.5 years (IQR: 2.0–8.0), and the median post-treatment interval was 50.0 months (IQR: 26.0–73.3). No statistically significant difference in questionnaire completion time was observed between the post-treatment CHD group and controls [405.00 (282.25–532.25) vs. 352.00 (248.25–463.75) seconds, *P* = 0.140].

According to the ESC-based CHD anatomic complexity classification, 27 (34.6%) participants had simple CHD, 41 (52.6%) had moderate-complexity CHD, and 10 (12.8%) had complex CHD. Thirty-three participants (42.3%) underwent minimally invasive occlusion procedures and 45 (57.7%) underwent cardiac surgery with cardiopulmonary bypass. The primary diagnosis distribution was VSD (38, 48.7%), ASD (16, 20.5%), PDA (0, 0.0%), TOF (4, 5.1%), DORV (5, 6.4%), and other CHD diagnoses (15, 19.3%).

### Internal consistency of the instruments

The internal consistency of the instruments in the current sample is shown in Table 2. Cronbach's alpha coefficients were 0.953 for the PedsQL 4.0 Generic Core Total Scale, 0.899 for the CES-DC, and 0.845 for the RSES. PedsQL domain alpha coefficients ranged from 0.818 to 0.905.

**Table 2:** Internal consistency of the PedsQL 4.0, CES-DC, and RSES in the current sample.

Scale	Domain/item set	Cronbach's *α*
PedsQL 4.0 Generic Core Total Scale	All items	0.953
PedsQL 4.0 Generic Core Scale	Physical Function	0.904
PedsQL 4.0 Generic Core Scale	Emotional Function	0.861
PedsQL 4.0 Generic Core Scale	Social Function	0.905
PedsQL 4.0 Generic Core Scale	School Function	0.818
CES-DC	All items	0.899
RSES	All items	0.845

CES-DC, Center for Epidemiologic Studies Depression Scale for Children; PedsQL 4.0, Pediatric Quality of Life Inventory 4.0 Generic Core Scales; RSES, Rosenberg Self-Esteem Scale.

### Scale score comparison

PedsQL 4.0 scores. Compared with controls, children and adolescents in the post-treatment CHD group had lower PedsQL 4.0 scores across all domains, including Physical, Emotional, Social, and School Functioning, as well as Physical Health, Psychosocial Health, and Total Scores (all *P* < 0.001; Table 3). Median PedsQL Total Score was 72.28 (IQR: 60.05–87.77) in the post-treatment CHD group and 89.67 (IQR: 77.45–95.65) in controls. In the age- and sex-adjusted linear regression model, controls had a higher PedsQL Total Score than post-treatment CHD patients (B = 13.31, 95% CI: 8.25–18.36, *P* < 0.001; Table 4).

**Table 3:** Scale scores for depressive symptoms, self-esteem, and HRQoL among post-treatment CHD patients and controls without known CHD.

Group	CES-DC	RSES	PedsQL 4.0 Generic Core Scale						
			Physical function	Emotional function	Social function	School function	Physical health	Psychosocial health	Total score
Post-treatment CHD	10.5 (3.25–17.75)	31.00 (28.00–36.75)	75.00 (59.38–87.50)	75.00 (60.00–90.00)	75.00 (70.00–98.75)	65.00 (50.00–85.00)	75.00 (59.38–87.50)	72.5 (58.75–89.58)	72.28 (60.05–87.77)
Controls without known CHD	8.0 (4.0–14.0)	36.00 (30.00–39.00)	93.75 (81.25–100.00)	90.00 (70.00–100.00)	100.00 (80.00–100.00)	82.5 (70.00–95.00)	93.75 (81.25–100.00)	88.33 (75.00–96.67)	89.67 (77.45–95.65)
*P* value	0.285	0.001	<0.001	<0.001	<0.001	<0.001	<0.001	<0.001	<0.001

Data are presented as median (interquartile range). *P* values were calculated using the Mann–Whitney *U*-test. CES-DC, Center for Epidemiologic Studies Depression Scale for Children; CHD, congenital heart disease; HRQoL, health-related quality of life; PedsQL 4.0, Pediatric Quality of Life Inventory 4.0 Generic Core Scales; RSES, Rosenberg Self-Esteem Scale.

**Table 4:** Age- and sex-adjusted linear regression models for PedsQL total score, CES-DC total score, and RSES total score.

Outcome	Variable	B	95% CI	*P* value
PedsQL total score	Group	13.31	8.25 to 18.36	<0.001
	Sex	−1.22	−6.27 to 3.83	0.634
	Age	−0.14	−1.01 to 0.73	0.751
CES-DC total score	Group	−2.62	−5.63 to 0.38	0.087
	Sex	0.19	−2.81 to 3.20	0.899
	Age	0.51	−0.01 to 1.03	0.054
RSES total score	Group	2.83	1.17 to 4.48	0.001
	Sex	0.25	−1.40 to 1.90	0.765
	Age	−0.25	−0.54 to 0.03	0.082

B represents the unstandardized regression coefficient. Group was coded as 0 for the post-treatment CHD group and 1 for controls without known CHD; therefore, a positive group coefficient indicates a higher outcome score among controls. Sex was coded as 0 for female and 1 for male. Age was entered as a continuous variable in years. CI, confidence interval; CES-DC, Center for Epidemiologic Studies Depression Scale for Children; CHD, congenital heart disease; PedsQL, Pediatric Quality of Life Inventory; RSES, Rosenberg Self-Esteem Scale.

Among post-treatment patients, PedsQL Total Scores tended to decrease with increasing CHD anatomic complexity, but no statistically significant difference was observed across simple, moderate-complexity, and complex CHD groups [80.43 (63.59–91.30), 71.74 (60.87–88.04), and 59.78 (49.46–77.45), respectively; *P* = 0.204; Table 5]. No statistically significant difference in PedsQL Total Score was observed between patients who underwent minimally invasive occlusion procedures and those who underwent cardiac surgery with cardiopulmonary bypass [80.43 (65.22–92.39) vs. 68.48 (58.70–85.87), *P* = 0.135]. When stratified by age at treatment, no statistically significant difference in PedsQL Total Score was observed between patients treated before 7 years of age and those treated at 7–18 years of age [75.00 (61.96–86.96) vs. 65.22 (55.43–89.13), *P* = 0.267].

**Table 5:** PedsQL 4.0 generic core scale scores by treatment strategy, CHD anatomic complexity, and age at treatment among post-treatment CHD patients.

PedsQL 4.0 Generic Core Scale	Physical function	Emotional function	Social function	School function	Physical health	Psychosocial health	Total score
Treatment strategy							
Minimally invasive occlusion procedure	81.25 (62.50–96.88)	75.00 (60.00–90.00)	85.00 (75.00–100.00)	70.00 (55.00–85.00)	81.25 (62.50–96.88)	80.00 (66.67–90.00)	80.43 (65.22–92.39)
Cardiac surgery with CPB	71.88 (56.25–81.25)	75.00 (60.00–85.00)	75.00 (65.00–90.00)	65.00 (45.00–85.00)	71.00 (56.25–81.25)	66.67 (55.00–88.33)	68.48 (58.70–85.87)
*P* value	0.079	0.507	0.160	0.285	0.079	0.259	0.135
CHD anatomic complexity							
Simple CHD	81.25 (62.50–93.75)	75.00 (55.00–100.00)	85.00 (75.00–100.00)	70.00 (52.50–85.00)	81.25 (62.50–93.75)	80.00 (64.17–90.00)	80.43 (63.59–91.30)
Moderate-complexity CHD	75.00 (59.38–87.50)	75.00 (60.00–85.00)	75.00 (70.00–100.00)	65.00 (50.00–85.00)	75.00 (59.38–87.50)	71.67 (60.00–88.33)	71.74 (60.87–88.04)
Complex CHD	60.94 (51.56–67.97)	65.00 (60.00–86.25)	75.00 (61.25–87.50)	42.50 (36.25–73.75)	60.94 (51.56–67.97)	60.83 (53.75–81.25)	59.78 (49.46–77.45)
*P* value	0.075	0.770	0.419	0.180	0.075	0.430	0.204
Age at treatment							
<7 years	75.00 (65.62–90.62)	75.00 (60.00–90.00)	75.00 (70.00–95.00)	70.00 (55.00–85.00)	75.00 (65.62–90.62)	75.00 (63.33–88.33)	75.00 (61.96–86.96)
7–18 years	62.50 (50.00–81.25)	75.00 (55.00–95.00)	75.00 (60.00–100.00)	55.00 (50.00–85.00)	62.50 (50.00–81.25)	66.67 (53.33–90.00)	65.22 (55.43–89.13)
*P* value	0.045	0.783	0.815	0.489	0.045	0.732	0.267

Data are presented as median (interquartile range). *P* values for two-group comparisons were calculated using the Mann–Whitney *U*-test, and *P* values across CHD anatomic-complexity groups were calculated using the Kruskal–Wallis test. CHD, congenital heart disease; CPB, cardiopulmonary bypass; PedsQL 4.0, Pediatric Quality of Life Inventory 4.0 Generic Core Scales.

CES-DC scores. Median CES-DC scores were numerically higher in the post-treatment CHD group than in controls, but no statistically significant between-group difference was observed [10.5 (3.25–17.75) vs. 8.0 (4.0–14.0), *P* = 0.285; Table 3]. The upper quartile in the post-treatment CHD group exceeded the commonly used screening threshold of 16, whereas the median score in both groups remained below this threshold.

In exploratory subgroup analysis, CES-DC scores appeared higher in the complex CHD subgroup [simple: 8.00 (2.00–17.00); moderate-complexity: 9.00 (4.00–14.00); complex: 19.00 (14.50–20.50); *P* = 0.047; Table 6]. However, this finding was based on only 10 complex cases and should be interpreted cautiously. No statistically significant difference in CES-DC scores was observed by treatment strategy [minimally invasive occlusion procedure: 6.00 (2.00–17.00); cardiac surgery with cardiopulmonary bypass: 13.00 (6.00–19.00); *P* = 0.080] or age at treatment [<7 years: 9.00 (3.00–17.00); 7–18 years: 13.00 (6.00–19.00); *P* = 0.337].

**Table 6:** CES-DC and RSES scores by treatment strategy, CHD anatomic complexity, and age at treatment among post-treatment CHD patients.

Subgroup	CES-DC	*P* value	RSES	*P* value
Treatment strategy				
Minimally invasive occlusion procedure	6.00 (2.00–17.00)	0.080	31.00 (28.00–36.00)	0.943
Cardiac surgery with CPB	13.00 (6.00–19.00)		31.00 (28.00–37.00)	
CHD anatomic complexity				
Simple CHD	8.00 (2.00–17.00)	0.047	32.00 (28.00–37.00)	0.840
Moderate-complexity CHD	9.00 (4.00–14.00)		31.00 (28.00–37.00)	
Complex CHD	19.00 (14.50–20.50)		30.00 (28.25–35.25)	
Age at treatment				
<7 years	9.00 (3.00–17.00)	0.337	34.00 (28.00–37.00)	0.207
7–18 years	13.00 (6.00–19.00)		31.00 (27.00–35.00)	

Data are presented as median (interquartile range). *P* values for two-group comparisons were calculated using the Mann–Whitney *U*-test, and *P* values across CHD anatomic-complexity groups were calculated using the Kruskal–Wallis test. CES-DC, Center for Epidemiologic Studies Depression Scale for Children; CHD, congenital heart disease; CPB, cardiopulmonary bypass; RSES, Rosenberg Self-Esteem Scale.

RSES scores. Median RSES scores were lower in the post-treatment CHD group than in controls [31.00 (28.00–36.75) vs. 36.00 (30.00–39.00), *P* = 0.001; Table 3]. This difference remained significant after adjustment for age and sex, with controls having higher RSES total scores (B = 2.83, 95% CI: 1.17–4.48, *P* = 0.001; Table 4). In exploratory subgroup analyses among post-treatment patients, no statistically significant differences in RSES scores were observed by CHD anatomic complexity [simple: 32.00 (28.00–37.00); moderate-complexity: 31.00 (28.00–37.00); complex: 30.00 (28.25–35.25); *P* = 0.840], treatment strategy (*P* = 0.943), or age at treatment (*P* = 0.207; Table 6).

In exploratory analyses among post-treatment CHD patients, post-treatment interval was not significantly correlated with PedsQL Total Score (*r* = 0.112, *P* = 0.330), CES-DC total score (*r* = −0.066, *P* = 0.565), or RSES total score (*r* = 0.088, *P* = 0.446).

## Discussion

In this online cross-sectional study, school-aged children and adolescents after treatment for CHD reported poorer HRQoL and lower global self-esteem scores than controls without known CHD, whereas no statistically significant difference in depressive symptom scores was observed. The reduction in HRQoL was evident across physical, emotional, social, and school functioning, and the between-group differences in PedsQL Total Score and RSES total score remained significant after adjustment for age and sex. These findings indicate that the longer-term burden after treatment for CHD in this sample extended across both daily functioning and global self-evaluation. This interpretation is consistent with broader evidence concerning psychological outcomes and interventions in individuals with CHD (19).

The HRQoL findings are consistent with both earlier and more recent evidence indicating persistent multidimensional limitations among children and adolescents with CHD during long-term follow-up (6, 9, 10, 16–18, 20). Reduced cardiopulmonary fitness may also contribute to limitations in physical functioning and participation among children with CHD (21), while multicenter controlled evidence has similarly demonstrated poorer quality of life in this population than in healthy peers (22). Importantly, the present pattern was multidimensional rather than purely physical. Lower emotional, social, and school functioning scores suggest that post-treatment challenges may include school absence, physical activity restriction, perceived fragility, delayed reintegration into peer activities, and protective attitudes from parents or teachers (5, 6). These issues may be particularly relevant in mainland China, where academic demands and family involvement in children's health and schooling are often substantial. Accordingly, our findings add to the still limited mainland Chinese evidence by indicating that recovery after treatment should be understood not only in biomedical terms, but also in relation to participation in everyday life.

The CES-DC results require careful interpretation. No statistically significant difference in median CES-DC scores was observed between groups, and the age- and sex-adjusted regression model likewise did not support a significant independent association between group status and CES-DC total score. Therefore, our data do not support a clear group-wide difference in depressive symptom burden after treatment for CHD. At the same time, the findings should not be read as indicating that psychosocial risk is absent. The upper quartile of the post-treatment CHD group exceeded the commonly used screening threshold of 16, suggesting that a subset of post-treatment patients may have elevated depressive symptoms. These results are better interpreted as evidence of heterogeneity in symptom burden rather than evidence of clinical depression. This distinction is important both methodologically and clinically.

The lower RSES scores in the post-treatment CHD group suggest that global self-esteem may also be affected after treatment for CHD. The post-treatment group had a lower median score, and the association remained significant after adjustment for age and sex. These scores were interpreted as continuous comparative measures because no universally accepted cutoff for low self-esteem has been established for Chinese children and adolescents using the scoring format applied in this study. Accordingly, the results indicate a group-level difference in global self-evaluation but should not be interpreted as a clinical classification of low self-esteem. Potential contributors include body-image concerns, medical dependence, activity limitations, and feeling different from peers, although the cross-sectional design and residual confounding prevent causal interpretation.

The exploratory subgroup findings should also be interpreted cautiously. Children with different CHD lesions may have markedly different trajectories after treatment in exercise capacity, neurodevelopment, academic performance, psychosocial functioning, and HRQoL. Outcomes after repair of ASD, VSD, or PDA may differ from those after TOF, DORV, or other complex defects. Although we reported both lesion-complexity categories and major diagnostic subtypes, the sample was too small for robust diagnosis-specific analyses. In exploratory subgroup analysis, CES-DC scores appeared higher in the complex CHD subgroup; however, this finding was based on only 10 complex cases and should be interpreted cautiously. Larger multicenter cohorts are needed to evaluate diagnosis-specific trajectories and to test whether time since treatment modifies HRQoL or psychosocial outcomes.

From a clinical perspective, these findings support a broader model of post-treatment follow-up. Long-term care for children and adolescents after treatment for CHD should extend beyond cardiac status alone and include routine assessment of HRQoL, school reintegration, physical activity guidance, family education, self-esteem, and other psychosocial concerns. This broader approach is consistent with recommendations promoting physical activity in individuals with CHD (23) and with general principles of cardiac rehabilitation (24). The present results are particularly relevant for school-aged patients, because poorer school and psychosocial functioning may have downstream implications for academic participation, peer relationships, and self-management during adolescence. Although no statistically significant group difference in CES-DC scores was observed, targeted psychosocial screening remains appropriate for children with complex lesions, school difficulties, marked activity restriction, repeated interventions, lower self-esteem, or ongoing family concern.

This study has several strengths. It focused specifically on school-aged children and adolescents after treatment for CHD, a clinically important group that remains underrepresented in mainland Chinese research. Online recruitment allowed inclusion of patients beyond face-to-face clinic encounters and may therefore better reflect part of the broader post-treatment community. The study also reported lesion-complexity categories, major diagnostic subtypes, treatment strategy, post-treatment interval, and internal consistency estimates for the instruments used. In addition, Chinese-language versions of the PedsQL 4.0, CES-DC, and RSES were applied, and age- and sex-adjusted regression analyses were performed for the major outcomes.

Several limitations should be acknowledged. First, the cross-sectional design does not permit conclusions regarding causality or temporal change. Second, data were collected through online self-report, which may have introduced selection bias and reporting bias, particularly among younger children who may have required assistance from guardians. Third, the post-treatment CHD group was recruited through WeChat-based follow-up groups, whereas controls were recruited through social media platforms. These different recruitment pathways may have introduced differences between groups beyond CHD status alone. Fourth, patients with major post-treatment complications, significant residual lesions, or reinterventions were excluded, so the study population likely represents children with relatively favorable outcomes after treatment. The findings should therefore be generalized cautiously to the broader population of children after treatment for CHD.

Additional limitations relate to measurement and confounding. Controls were identified on the basis of reported health history rather than clinical examination or echocardiographic screening, so asymptomatic or undiagnosed CHD could not be fully excluded. Important determinants of HRQoL and mental health, including socioeconomic status, parental education, parental occupation, family functioning, urban-rural residence, school context, physical activity level, and geographic region, were not collected or adjusted for. Although exploratory correlation analyses did not identify a clear association between post-treatment interval and the major outcomes, the cross-sectional design and limited sample size precluded formal evaluation of post-treatment duration as an effect modifier. In addition, RSES item 2 was inadvertently presented with negative rather than standard positive wording. It was scored according to the wording actually administered, and the corrected 10-item scale showed good internal consistency; nevertheless, this wording departure may limit direct comparison with studies using the standard item wording. Finally, subgroup analyses were limited by sample size, especially in the complex CHD subgroup, and should therefore be regarded as exploratory. Future research should use multicenter longitudinal designs to examine trajectories of HRQoL and psychosocial adjustment after treatment for CHD, identify context-specific risk factors in Chinese populations, and evaluate interventions that support physical activity, school participation, and family adaptation.

In conclusion, school-aged children and adolescents after treatment for CHD in mainland China reported poorer HRQoL and lower global self-esteem scores than controls without known CHD, whereas no statistically significant difference in depressive symptom scores was observed. These findings highlight the need to extend post-treatment care beyond cardiac status and to address functional recovery, school participation, family education, physical activity guidance, and psychosocial support.

## Data Availability

The raw data supporting the conclusions of this article will be made available by the authors, without undue reservation.
